# Safety and efficacy of intravenous thrombolytic treatment in wake‐up stroke: Experiences from a single center

**DOI:** 10.1002/brb3.2152

**Published:** 2021-05-03

**Authors:** Adam Wiśniewski

**Affiliations:** ^1^ Department of Neurology Faculty of Medicine Collegium Medium in Bydgoszcz, Nicolaus Copernicus University in Toruń Bydgoszcz Poland

**Keywords:** alteplase, efficacy, safety, thrombolysis, unclear onset, Wake‐up stroke

## Abstract

**Objectives:**

Wake‐up stroke is an important clinical problem that may account for a quarter of all ischemic strokes. This study aimed to establish the safety and efficacy of intravenous thrombolytic treatment of wake‐up strokes by comparing it to the standard thrombolysis treatment in strokes with clear onsets and wake‐up strokes that did not receive reperfusion therapy.

**Methods:**

This retrospective study enrolled 95 patients with ischemic strokes who underwent thrombolytic treatment with alteplase, including nine patients with wake‐up strokes. The safety profile (mortality and intracranial bleeding) and efficacy (clinical and functional outcomes on admission, discharge, and 90 days after stroke onset) were evaluated.

**Results:**

When assessed using the modified Rankin scale (mRs), the patients with wake‐up strokes had significantly more favorable functional outcomes on discharge when compared to those who received standard thrombolysis (*p* = .0289). No significant differences were noted when the favorable outcome rate (mRs score = 0–2) at three months post‐thrombolysis (Odds ratio [OR] = 2.07; 95% confidence interval [CI] = 0.41–10.6; *p* = .3807) and safety outcomes (death during hospitalization: OR = 0.49; 95% CI = 0.03–9.11; *p* = .6295 and intracranial bleeding 24 hr after treatment: OR = 0.43; 95% CI = 0.02–7.58; *p* = .5707) were compared between the two groups. The Cochran–Mantel–Haenchel shift analysis showed a significantly more favorable distribution of the mRs scores at three months after the stroke onset in the patients with wake‐up strokes who were treated with alteplase compared to those who did not receive thrombolysis (OR = 1.42; 95% CI = 1.01–1.82; *p* = .0426).

**Conclusions:**

Our study demonstrated that in patients who awaken with stroke symptoms, intravenous thrombolytic treatment is a safe procedure that may lead to favorable outcomes. Further studies should be performed to increase the size of the group of patients with wake‐up strokes who can be treated with reperfusion therapy.

## Graphical abstract

The aim of the study was to establish the safety and efficacy of intravenous thrombolytic treatment in wake‐up stroke comparing to the standard thrombolysis treatment in stroke with clear onset. Wake‐up stroke subjects were characterized by significantly more favorable functional outcome on discharge in contrast to the standard thrombolysis procedure subjects (*p* = .0289). Our study showed that intravenous thrombolytic treatment is a safe procedure and may lead to a favorable outcome in subjects who are awaken with stroke symptoms.

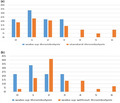

## 1 INTRODUCTION

Wake‐up stroke is a pertinent clinical problem that accounts for a quarter of all ischemic strokes (Mackey et al., 2011). Until recently, patients who woke up with stroke symptoms had no specific therapeutic options, making them a group that is at high risk of disability (Denny et al., 2014). The wake‐up clinical trial (Thomalla et al., 2018) was a breakthrough in this field. It enabled a select group of patients to receive intravenous thrombolytic therapy. The primary inclusion criteria were as follows: age <80 years old, premodified Rankin scale (mRs) 0–1 points, National Institute of Health Stroke Scale (NIHSS) <25 points, high risk of disability, and duration of wakefulness below 4.5 hr. The contraindications included any general contraindication to intravenous thrombolysis, thrombectomy candidates, patients with large ischemic strokes (diffusion‐weighted imaging [DWI] changes in more than one‐third of the middle cerebral artery (MCA) territory and more than a half of the anterior or posterior cerebral artery territory). All the patients had an acute ischemic lesion that was visible on diffusion‐weighted magnetic resonance imaging (MRI); however, they did not have clear parenchymal hyperintensity on fluid‐attenuated inversion recovery (FLAIR)—also known as “DWI‐FLAIR mismatch”—that indicated that the stroke had approximately occurred within the preceding 4.5 hr (Thomalla et al., 2011). A favorable outcome at three months after the stroke onset and lower median mRs score were reported significantly more often in the group that was treated with alteplase. Moreover, the group that was treated with alteplase demonstrated neither a higher mortality nor a higher rate of symptomatic intracranial bleeding when compared to patients who did not receive thrombolytic treatment. Based on these results, the updated guidelines for the management of ischemic stroke by the American Heart Association/American Stroke Association allowed the administration of alteplase within 4.5 hr of the recognition of stroke symptoms in patients with acute ischemic strokes who (1) awoke with stroke symptoms or had an unclear time of onset that was >4.5 hr from their last known well or baseline state and (2) who had a DWI‐MRI lesion that was smaller than one‐third of the MCA territory and that had no visible signal (Powers et al., 2018).

The aim of this study was to establish the safety and efficacy of thrombolytic treatment in a wake‐up stroke protocol based on the experiences of a single‐center stroke intensive care unit by comparing it to the standard thrombolysis treatment procedure in strokes with clear onsets.

## 2 MATERIALS AND METHODS

This study was a retrospective, single‐center, case series. Data from 95 ischemic stroke patients who underwent intravenous thrombolytic treatment with alteplase between 1 January 2019 and 30 September 2020 were analyzed. Of these patients, nine were treated using the wake‐up stroke protocol, while the remaining patients were treated according to the standard procedure. The inclusion criteria for reperfusion therapy in patients with wake‐up strokes were implemented in accordance with Polish guidelines. The guidelines stipulated that, in general, intravenous thrombolytic treatment could be performed when ischemic changes were clearly visible in DWI and were, simultaneously, not clearly visible on FLAIR (similar to the original trial),) and when no more than 4.5 hr had passed since the patient awoke with stroke symptoms. Additionally, we adhered to the inclusion and exclusion criteria that were used in the original trial (e.g., we. w excluded patients who also underwent mechanical thrombectomy). Clinical outcomes were measured using the NIHSS, both on admission and discharge. The patients’ functional conditions and outcomes were measured using the mRs, on admission, on discharge, and three months after the stroke onset. Follow‐up cerebral neuroimaging was performed 24 hr after thrombolysis to investigate the intracranial hemorrhage detectability. The safety profile included death during hospitalization or intracranial bleeding on the control neuroimaging. This study was approved by the Bioethics Committee of Nicolaus Copernicus University in Torun at Collegium Medicum of Ludwik Rydygier in Bydgoszcz (KB number 459/2020). The nonparametric Mann–Whitney *U* test was used to analyze continuous variables. Categorical variables were analyzed using the chi‐square test. Logistic regression analysis was used to analyze the safety and efficacy outcomes. The Cochran–Mantel–Haenchel shift analysis test was used to compare the mRs score distributions between the selected groups at three months after the stroke onset. Statistical significance was defined as *p* < .05.

## 3 RESULTS

Overall, between 1 January 2019 and 30 September 2020, there were nine cases of intravenous thrombolysis that were treated using the wake‐up stroke protocol. In five patients, the final qualification for thrombolysis was controversial and extension of the wake‐up protocol was required after the patient's consent was obtained. This included providing comprehensive information about the possible benefits and risks of thrombolysis. Detailed descriptions of these cases are provided below. The remaining patients, who are not mentioned below, met all the inclusion and exclusion criteria that corresponded to the original trial and the DWI/FLAIR mismatch did not raise any doubts.

### 3.1 Case 1

A 44‐year‐old man was admitted to the hospital with right limb hemiparesis and dysarthria. On admission, the NIHSS and mRs scores were 8 and 4, respectively. He awoke with stroke symptoms at 05.00 AM and was admitted to hospital at 08.30 AM. He had a history of hypertension and tramadol, heroine, amphetamine abuse. Following the blood investigations and computed tomography (CT) scan, MRI with DWI and FLAIR sequences was performed. This is where the mismatch was noticed. The patient was admitted to the stroke intensive care unit at 09.28 AM with a blood pressure of 205/110 mmHg; therefore, a decision to simultaneously administer alteplase and a continuous intravenous urapidil infusion was made. Despite the use of a hypotensive drug, the systolic blood pressure averaged between 180 mmHg and 210 mmHg. Nonetheless, the patient received the full alteplase dose (90 mg). Ultimately, the patient had no hemorrhage on the control neuroimaging and was discharged with NIHSS and mRs scores of 6 and 3, respectively.

### 3.2 Case 2

**FIGURE 1:**
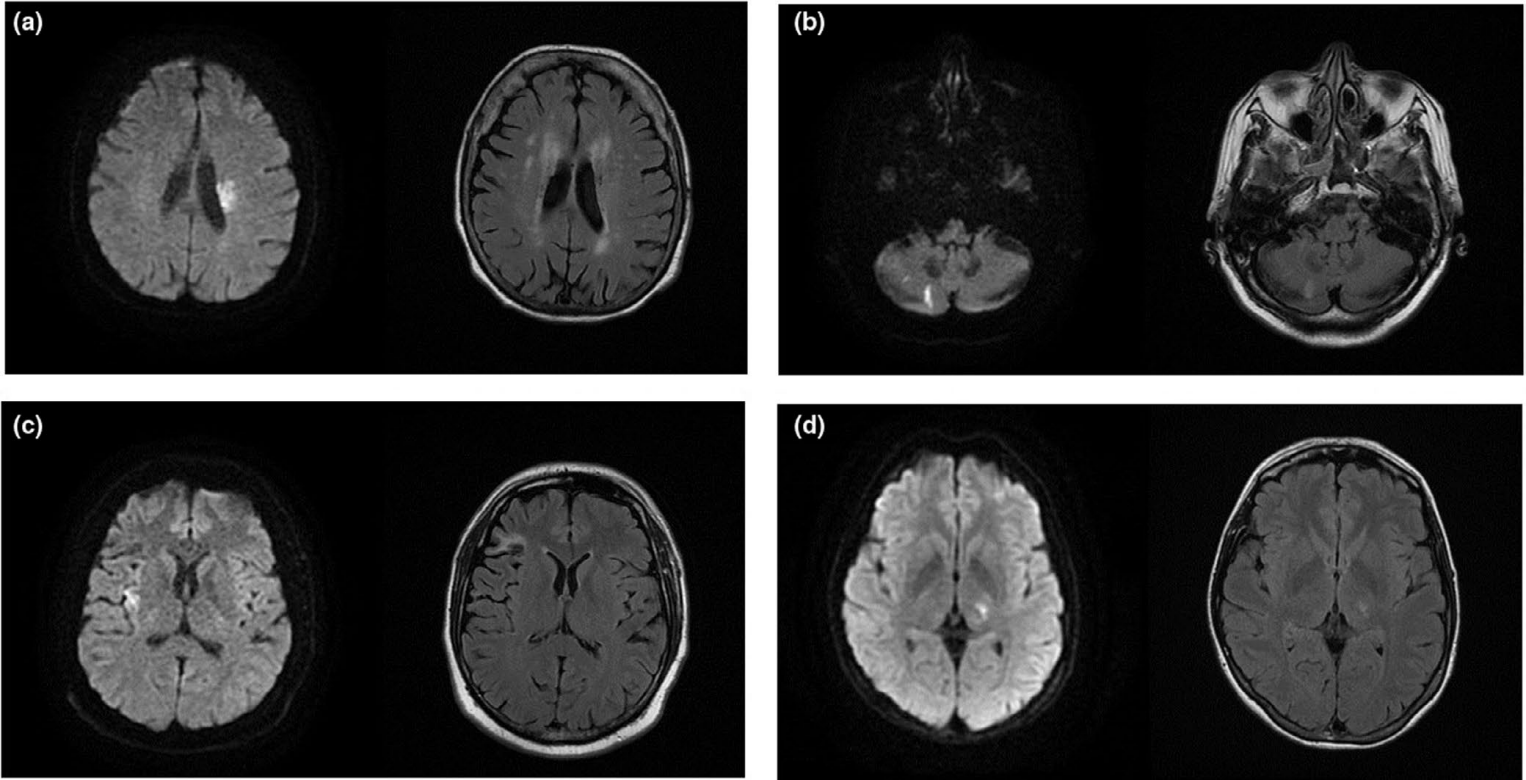
Diffusion‐weighted imaging (DWI) and fluid‐attenuated inversion recovery (FLAIR) mismatch in the selected cases of wake‐up stroke. (a) The clear DWI/FLAIR mismatch (left side) presented in case 2. (b) The acute ischemic lesions visualized 24 hr post‐thrombolysis on DWI (left side) and FLAIR in case 3. (c) The clear DWI/FLAIR mismatch (left side) presented in case 4. (d) The doubtful DWI/FLAIR mismatch (left side) presented in case 5

An 87‐year‐old woman was admitted to the hospital with right limb hemiparesis. On admission, the NIHSS and mRs scores were 6 and 3, respectively. She awoke with stroke symptoms at 04.30 AM and was admitted to the stroke unit at 07.00 AM. She had a history of hypertension. Following the blood investigations and CT scan, MRI with DWI and FLAIR sequences was performed. This is where the mismatch was noticed (Figure 1a). The patient was administered 68 mg of alteplase. No hemorrhage was visualized on the control CT scan. She was discharged with NIHSS and mRs scores of 4 and 2, respectively.

### 3.3 Case 3

A 76‐year‐old woman was admitted to the hospital with symptoms of a brainstem stroke (peripheral left facial nerve damage, transient left limb hemiparesis, left‐sided ataxia, right‐sided facial hemianesthesia, dysarthria, right‐sided Horner's syndrome, and right‐sided nystagmus). On admission, the NIHSS and mRs scores were 7 and 4, respectively. The patient awoke with stroke symptoms at 04.00 AM and was last seen without symptoms at 10.00 p.m. the previous day. She was admitted to the stroke unit at 07.00 AM. She had a history of hypertension. Following the blood investigations and CT scan, MRI with DWI and FLAIR sequences was performed, where no acute ischemic lesions were noticed. However, upon recognizing the clinical diagnosis of an ischemic stroke and excluding other causes, considering that there were no changes to the FLAIR, we decided to administer 56 mg of alteplase and perform a control MRI after 24 hr. We were able to visualize acute ischemic changes in the cerebellum, both on DWI and FLAIR. No hemorrhagic transformation was detected (Figure 1b). The patient 1B was discharged with NIHSS and mRs scores of 2 and 1, respectively.

### 3.4 Case 4

A 83‐year‐old man was admitted to the hospital with left limb hemiparesis and dysarthria. On admission, the NIHSS and mRs scores were 8 and 3, respectively. The patient awoke with stroke symptoms at 05.30 AM and was admitted to hospital at 08.30 AM. He had a history of hypertension and smoking. Following the blood investigations and CT scan, MRI with DWI and FLAIR sequences was performed and a clear mismatch was noticed (Figure 1c). The patient was administered 1C 81 mg of alteplase. He was discharged with NIHSS and mRs scores of 3 and 1, respectively.

### 3.5 Case 5

A 34‐year‐old woman was admitted to the hospital with right‐sided hemiparesis and hemianesthesia. On admission, the NIHSS and mRs scores were 6 and 3, respectively. She awoke with stroke symptoms at 07.00 AM, was last seen without symptoms at 11.00 p.m. the preceding day, and was admitted to the stroke unit at 10.00 a.m. She had a history of hyperlipidemia and oral hormonal contraceptive use. Following the blood investigations and CT scan, MRI with DWI and FLAIR sequences was performed. We were doubtful as to whether there was a mismatch present (Figure 1d); however, 1D the radiologist identified an evident focus on DWI that corresponded to a faintly distinct hyperintense focus on FLAIR. Due to the patient's young age, the increased risk of disability, and the lack of any clearly visible changes on FLAIR, after obtaining the patient's consent, we administered 57 mg of alteplase. No intracranial bleeding was shown in the control CT and she was discharged with NIHSS and mRs scores of 2 and 1, respectively. During the patient's hospital stay, a persistent foramen ovale was noted. It was closed two months later using an occluder.

Overall, 17 patients prequalified for treatment according to the protocol. Ultimately, eight of these patients were excluded due to obvious exclusion background (including 6 patients who did not have a mismatch and 2 who had extensive areas of ischemia). There were no incidents of intracranial bleeding in the control neuroimaging that was performed 24 hr after alteplase administration. None of the patients mentioned above had large intracranial vessel occlusions on CT brain angiography; therefore, they were not candidates for mechanical thrombectomy. During the study period, a total of 43 patients had stroke symptoms upon waking. However, 26 of these patients did not qualify for further diagnosis using the wake‐up protocol (21 arrived after 4.5 hr and 5 had hemorrhagic strokes on CT). Ultimately, this meant that only 21% of the patients with wake‐up strokes received intravenous thrombolysis.

**FIGURE 2:**
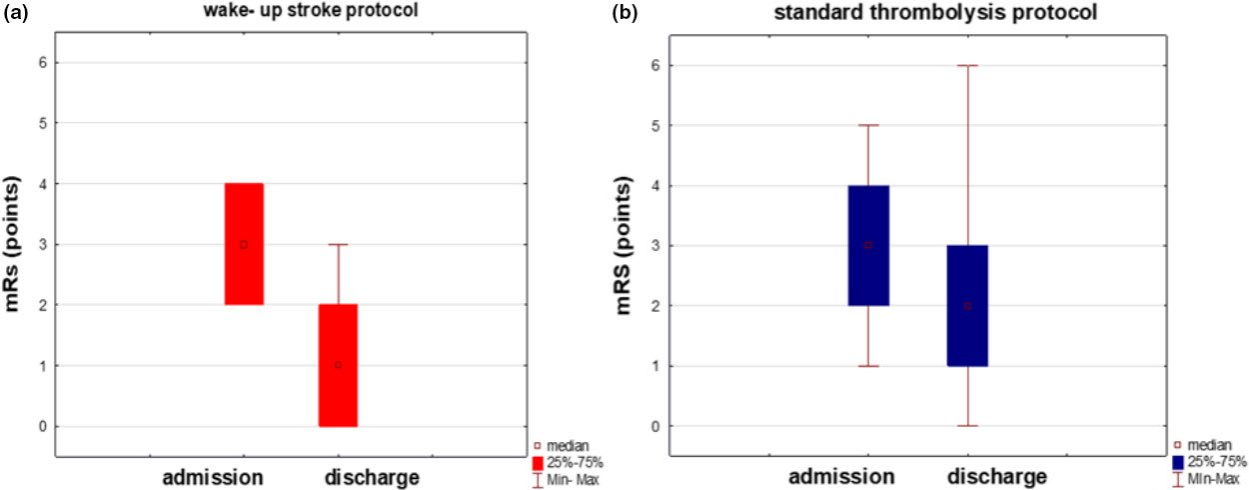
Comparison of the functional conditions (estimated using the modified Rankin scale) on admission and at discharge in the patients who received the wake‐up stroke protocol and those with clear stroke onsets who received the standard thrombolysis protocol. mRs: modified Rankin scale

**FIGURE 3:**
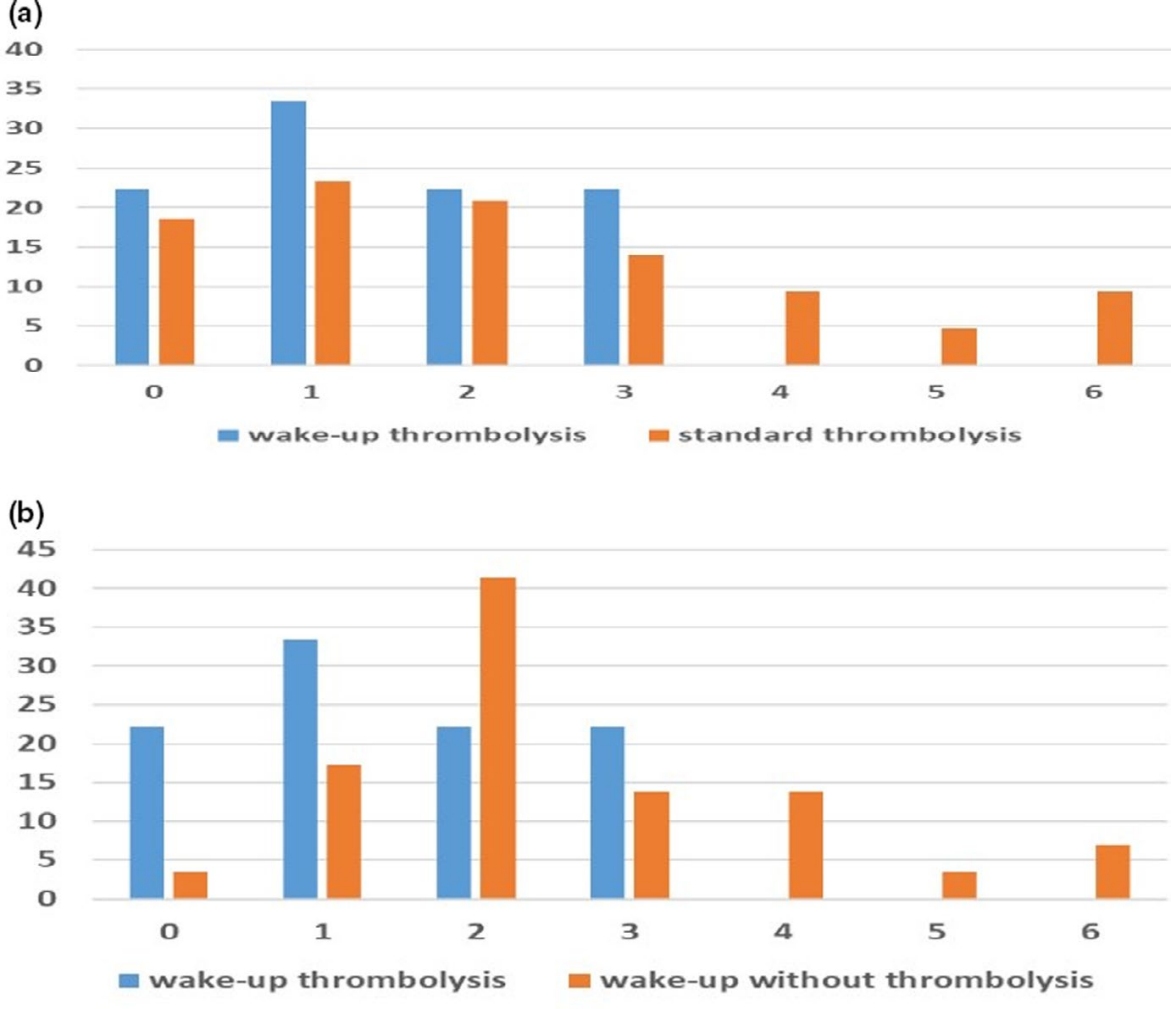
The percentage distribution of the modified Rankin scale levels (mRs 0–6) at three months after the stroke onset. (a) No significant differences in the functional outcomes were noted when the patients who received the wake‐up stroke protocol were compared to those who received the standard protocol. (b) The significant shift between the patients with wake‐up strokes that underwent reperfusion therapy and those who did not receive thrombolysis treatment

Table 1 shows the stroke patients’ general clinical characteristics and compares the patients who received the wake‐up and standard protocols for reperfusion therapy. It is worth mentioning that the patients with wake‐up strokes had significantly more favorable functional outcomes (as estimated using the mRs scores) on discharge when compared to the patients who received the standard thrombolysis procedure, who had comparable scores on admission (Figure 2). At three months after the stroke onset2, there were no significant differences noted when the favorable outcome rates of the two groups were compared (mRs score = S0–1, odds ratio [OR] = 1.73, 95% confidence interval [CI] = 0.44–6.92, *p* =.4343; mRs score = S0–2, OR = 2.07, 95% CI = 0.41–10.6, *p* = .3807). In addition, in the shift analysis, no significant differences in the distribution of the mRs scores at three months after the stroke onset were noted when the two groups were compared (OR = 1.12, 95% CI = 0.87–1.45, *p* =.1124; Figure 3a). Logistic regression analysis also demonstrated no significant differences in safety outcomes when the two groups were compared (death during hospitalization: OR = 0.49, 95% CI = 0.03–9.11, *p* = .6295; intracranial bleeding 24 hr after receiving treatment: OR = 0.43, 95% CI = 0.02–7.58, *p* = .5707).

**TABLE 1:** The general characteristics of the stroke patients who received thrombolysis and a and comparison of the patients with wake‐up strokes and those who received standard protocol treatment

Parameter	Wake‐up stroke thrombolysis, *N* = 9	Standard intravenous thrombolysis, *N* = 86	p‐values
Age, median (range)	63.4 (34–87)	67.2 (36–99)	0.2741
Sex, male, *N* (%)	4 (44%)	42 (48.8%)	0.8019
Hypertension, *N* (%)	8 (88.9%)	78 (90.7%)	0.8965
Diabetes, *N* (%)	4 (44.5%)	38 (44.2%)	0.9655
Hyperlipidemia, *N* (%)	4 (44.5%)	28 (32.6%)	0.7898
Smoking, *N* (%)	3 (33.3%)	32 (37.2%)	0.8965
Systolic pressure (admission), median (range)	168 (124–205)	166 (120–235)	0.8653
Diastolic pressure (admission), median (range)	96 (68–110)	98 (64–124)	0.8214
Cardioembolism, *N* (%)	3 (33.3%)	31 (36%)	0.7689
Large vessel disease, *N* (%)	2 (22.2%)	16 (18.6%)	0.6324
Small vessel disease, *N* (%)	4 (44.5%)	32 (37.2%)	0.4368
Time to thrombolysis after stroke onset in minutes, median (range)	235 (168–270)	221 (72–270)	0.7842
Alteplase dose (mg) median (range)	76.4 (56–90)	74.2 (45–90)	0.7412
Intracranial hemorrhage in control scan, *N* (%)	0 (0%)	9 (10.4%)	0.2890
NIHSS score on admission (points) median (range)	5 (3–8)	5 (1–24)	0.4975
NIHSS score at discharge (points), median (range)	3 (1–7)	4 (1–32)	0.3120
Early neurological improvement (decrease in the NIHSS score by at least 2 points), *N* (%)	5 (55.6%)	28 (32.6%)	0.1618
Early neurological deterioration (increase in the NIHSS score by at least 2 points), *N* (%)	0 (0%)	11 (12.8%)	0.2539
mRs score on admission, median (range)	3 (2–4)	3 (1–5)	0.7660
mRs score at discharge, median (range)	1 (0–3)	2 (0–6)	0.0289
A favorable outcome at three months post‐thrombolysis			
mRs score=0–1	5 (55.5%)	36 (41.8%)	0.4300
mRs score=0–2	7 (77.7%)	54 (62.8%)	0.3722
Death during hospitalization, *N* (%)	0 (0%)	8 (9.3%)	0.3390

Note

Continuous variables were assessed using the Mann‐Whitney *U* test. Categorical variables were assessed using the Chi‐square test.

Abbreviations: mRs, modified Rankin scale; NIHSS, National Institutes of Health Stroke Scale.

Table 2 shows the comparison of the clinical outcomes for the patients with wake‐up strokes who were treated with alteplase and those who did not receive thrombolysis. Patients who underwent reperfusion therapy had significantly lower total mRs scores on discharge and were more likely to exhibit excellent functional outcomes (mRs score=(mRS0–1) at three months after the stroke onset compared to the patients who did not undergo thrombolysis (OR = 4.79, 95% CI = 0.97–23.5, *p* = .0438). Moreover, the shift analysis showed a significantly more favorable distribution of mRs scores at three months after the stroke onset in the group that was treated with alteplase compared to the group that was not (OR = 1.42, 95% CI = 1.01–1.82, *p* =.0426; )‐Figure 3b).

**TABLE 2:** Clinical outcome comparison of the patients with wake‐up strokes who were treated with alteplase and those who did not receive reperfusion therapy

Parameter	Wake‐up stroke with thrombolysis, *N* = 9	Wake‐up stroke with no reperfusion therapy, *N* = 29	p‐values
NIHSS score on admission (points) median (range)	5 (3–8)	6 (3–22)	0.2456
NIHSS score at discharge (points), median (range)	3 (1–7)	4 (1–28)	0.4246
Early neurological improvement (decrease in the NIHSS score by at least 2 points), *N* (%)	5 (55.6%)	8 (27.6%)	0.1618
Early neurological deterioration (increase in the NIHSS score by at least 2 points), *N* (%)	0 (0%)	6 (20.7%)	0.2539
mRs score on admission, median (range)	3 (2–4)	3 (1–5)	0.6850
mRs score at discharge, median (range)	1 (0–3)	2 (0–6)	0.0318
A favorable outcome at three months post‐thrombolysis			
mRs score=0–1	5 (55.5%)	6 (20.7%)	0.0439
mRs score=0–2	7 (77.7%)	18 (62.1%)	0.4246
Death during hospitalization, *N* (%)	0 (0%)	2 (6.9%)	0.4890

Note

Continuous variables were assessed using the Mann‐Whitney U test. Categorical variables were assessed using the Chi‐square test.

Abbreviations: mRs, modified Rankin scale; NIHSS, National Institutes of Health Stroke Scale.

## 4 DISCUSSION

The timing of the onset of strokes is not distributed evenly over the 24 hr in a day (Elfil et al., 2019). Similar to myocardial infarctions, ischemic strokes are more common in the morning. The mechanisms that play a leading role in this phenomenon include the sympathetic nervous system activation, catecholamine burst, increased platelet aggregation, and raised levels of prothrombotic factors in the morning (Andrews et al., 1996; Somers et al., 1993). It is estimated that approximately 58% of all ischemic strokes may occur between 06.00 AM and 09.00 PM. Taking the above period of increased risk into account, studies have proposed that the onset of strokes usually occurs at or near the time of awakening (Elliott, 1998). These data became the basis for the attempts to undertake specific treatment measures that would provide an opportunity for patients to avoid disability. However, despite the updated guidelines and increasingly common diagnostic options in neuroimaging, including perfusion and diffusion neuroimaging (Fink et al., 2002), there are still little data on the safety and efficacy of intravenous alteplase or mechanical thrombectomy in wake‐up stroke.

Ahmed et al. (2020) demonstrated that most patients (65.2%) who were treated using a wake‐up protocol that included alteplase and that was based on DWI/FLAIR mismatch exhibited a favorable functional outcomes 90 days after thrombolysis (mRs score = 0–1). A study by Aoki et al. (2011) that was based on DWI/FLAIR mismatch found no incidence of symptomatic hemorrhage and a moderate rate (40%) of asymptomatic hemorrhage among patients with wake‐up strokes who were treated with alteplase. AA favorable outcome (mRs score = 0–2) at three months after the stroke onset was reported in 40% of their study participants, % while a favorable outcome on discharge in 30% of their study participants. Through multivariate logistic regression analysis, Kim et al. (2011) found that intravenous thrombolysis was an independent factor for favorable outcomes at three months after the stroke onset and that intravenous thrombolysis was not related to a higher risk of intracranial bleeding when compared to nonthrombolyzed patients. However, their study sample included all stroke patients with unclear onsets and only had a wake‐up stroke rate of 33%. In a study by Breuer et al. (2010), the screening feasibility rate for thrombolysis among patients with wake‐up strokes was 22%. This finding was consistent with our results. In their study, the patient's ability to qualify for intravenous thrombolysis was based on the presence of perfusion‐weighted imaging (PWI)/DWI mismatch. Favorable outcomes were reported at three months after the stroke onset in 30% (mRs score = 0–1) and 50% of the participants (mRs score=0–2). They achieved a low rate of asymptomatic intracranial bleeding (10%) and did not detect any cases of symptomatic hemorrhage. Cho et al. (2008) used both PWI/DWI and DWI/FLAIR mismatches and compared patients with unknown stroke onset times (including those with wake‐up strokes) who were treated with alteplase to those who had known stroke onset times that were treated with standard thrombolysis. They did not report any significant differences in the clinical outcomes (mRs score = 0–2 in 50% of the patients after three months) and safety endpoints (6.3% symptomatic intracranial hemorrhage rate) when the two groups were compared.

Through the use of CT‐based data (excluding large ischemic lesions and intracranial bleeding), Barreto et al. (2016) also demonstrated favorable outcomes (mRs score = 0–1) after three months in 52.6% of the patients with wake‐up strokes who were treated with alteplase (administered within three hours of awakening with stroke symptoms) and there were no cases of symptomatic hemorrhage reported (the asymptomatic hemorrhage rate was 15%). Cortijo et al. (2013) used CT perfusion to make decisions regarding thrombolytic therapy. Favorable outcomes were reported in 56.3% of the patients (mRs score = 0–2) at 90 days postintervention, and there were no cases of symptomatic hemorrhage. The asymptomatic hemorrhage rate was moderate (21.9%). Campbell et al. (2019) conducted a meta‐analysis and demonstrated that extension of the thrombolysis time to nine hours (including in patients with wake‐up strokes) based on perfusion imaging was associated with excellent functional outcomes (mRs score = 0–1) at three months after the stroke onset when compared to a placebo (36% versus 29%). However, the incidence of symptomatic intracranial bleeding was higher, and the mortality rate was not significantly increased in alteplase group when compared to a placebo. Mourand et al. (2015) used the DWI/FLAIR mismatch criteria to evaluate the safety and efficacy of thrombolysis and bridging therapy (thrombolysis followed by mechanical thrombectomy) in patients with wake‐up strokes. The authors revealed that 52% of patients with wake‐up strokes experienced favorable outcomes (mRs score = 0–2) at three months post‐thrombolysis. Moreover, they demonstrated that combined therapy may be more effective and lead to significantly better outcomes (61% of the patients had an mRs score = 0–2) and lowered rates of mortality (7.3%) and symptomatic intracranial hemorrhage (4.9%).

Another therapeutic option that is still being researched is tenecteplase. It is a modified alteplase molecule with higher specificity to fibrin and a better pharmacokinetic properties, including a faster onset of action and longer half‐life than alteplase (Dunn & Goa, 2001; Tsikouris & Tsikouris, 2001). According to the afore‐mentioned data, it is thought that its use for thrombolysis may prove to be more effective and safer that alteplase (Logallo et al., 2017). A study by Ahmed et al. (2020) that compared tenecteplase to alteplase for wake‐up stroke thrombolysis showed no significant difference in the favorable outcomes after three months and safety profile when the two were compared. The Tenecteplase in Wake‐up Ischaemic Stroke Trial (2017) which is currently ongoing, randomizes patients who awoke with stroke symptoms to receive treatment with 0.25 mg/kg tenecteplase based only on their CT scan (ClinicalTrials: NCT03181360). The use of CT alone may increase the frequency of thrombolysis treatment and shorten the door‐to‐needle time. The clinical trial promises to increase the percentage of patients who may benefit from specific therapies.

In terms of safety and effectiveness, the results obtained in this study were consistent with other reports on this topic. However, in most cases, the current diagnostic and treatment guidelines were followed. In special, distinguished cases, while taking into account the ratio of potential benefits to possible risks and always with the patient's consent, a decision was made to extend the range of indications for reperfusion treatment (older age, no changes in DWI, or a questionable mismatch). The results of the modified off‐label therapy were positive. There were no cases of clinical deterioration or secondary intracerebral bleeding as an adverse effect of intravenous alteplase treatment reported. Furthermore, most of the patients benefited from the treatment and this translated into lower NIHSS and mRs scores. Moreover, a novel finding, that had not yet been reported, was also emphasized in this study. There were more favorable functional outcomes on discharge in the patients with wake‐up strokes when compared to standard thrombolysis procedures in patients who had a certain time of stroke onset. Our observation of this short‐term improvement in the functional condition was surprising and may have been indicative of the advantages of using MRI to assess whether patients of qualified for reperfusion treatment compared to CT. However, ultimately, the outcomes at discharge did not translate into more favorable long‐term prognoses, both in dichotomous and shift statistics, compared to the standard thrombolysis procedure. This significantly impairs the importance of this finding. Moreover, the comparable data on the risk of death and intracranial bleeding in our study that was consistent with other studies were indicative of the high safety profile of thrombolysis in wake‐up strokes. Higher rates of favorable outcomes at three months after the stroke onset compared to other studies could be explained by the fact that the treatment was generally administered to patients with good baseline clinical conditions.

It is notable that both the dichotomous approach and ordinal shift analysis demonstrated that patients with wake‐up strokes that who received thrombolytic treatment achieved more favorable long‐term functional outcomes compared to the patients who did not receive subjects alteplase. The above data prove the effectiveness of reperfusion therapy with this particular type of stroke. However, studies have not yet shown that thrombolysis can significantly increase the proportion of stroke patients with early neurological improvement. This may indicate that thrombolysis improves long‐term prognosis and does not cause significant fluctuations in the clinical status in the acute stage of stroke.

Nevertheless, due to the limitations of this study, the above findings should be approached with caution. These limitations included the fact that this study had a small sample size and was a nonrandomized retrospective analysis that was based on a single‐center's results. Further studies that analyze the capacity of the inclusion criteria to the increased number of potentially curable patients who awaken with stroke symptoms are required.

Despite the development of new therapeutic options, wake‐up stroke still remains a significant clinical challenge, because less than a quarter of the patients can undergo reperfusion therapy. Our study and the data from the literature established that thrombolytic treatment is a safe procedure that could lead to favorable outcomes in patients who are awaken with stroke symptoms. Research should strive to expand the indications for safe treatment to include as many stroke patients as possible and to allow for the provision of specific treatment that will reduce the risk of disability. However, there is still controversy around the choice of neuroimaging methodology that should be used to determine whether patients qualify for thrombolysis and the duration of the therapeutic window.

## CONFLICT OF INTEREST

None declared.

### PEER REVIEW

The peer review history for this article is available at https://publons.com/publon/10.1002/brb3.2152.

## Data Availability

The author confirms that the data supporting the findings of this study are available within the article.
